# The plastome of *Phaius hainanensis* (Orchidaceae): an endangered species endemic to Hainan province, China

**DOI:** 10.1080/23802359.2021.1904801

**Published:** 2021-03-26

**Authors:** Lu Tang, Han-qing Tang, Yan Luo, Li-Ping Ge

**Affiliations:** a College of Forestry, Shanxi Agricultural University, Jinzhong, PR China; b Department of Gardening and Horticulture, Xishuangbanna Tropical Botanical Garden, Chinese Academy of Sciences, Yunnan, PR China; c Department of Gardening and Horticulture, Core Botanical Gardens, Chinese Academy of Sciences, Yunnan, PR China

**Keywords:** Orchidaceae, *Phaius hainanensis*, plastome, phylogenomics

## Abstract

*Phaius hainanensis* C. Z. Tang et S. J. Cheng is a species with extremely small populations and is endemic to China. Genetic data of this orchid species is minimal. With the aim to identify appropriate chloroplast markers for the use in conservation biology studies, the plastome of *P. hainanenisis* was assembled. The plastome of *P. hainanensis* is 158,314 bp in length and contains a large single copy region of 86,700 bp in length, a small single copy region of 18,452 bp, and a pair of inverted repeats of 26,581 bp. The annotation predicted 114 unique genes, including 80 protein-coding, 30 *tRNA*s, and four *rRNA*s. Seventeen genes contained a single intron and two genes (*clpP* and *ycf3*) have two introns. The GC content of *P. hainanensis* is 36.9%. Phylogenetic analysis indicated *P. hainanensis* is closely related to *P. tancarvilleae,* and it also supported that *Phaius* and *Calanthe* are sister groups. The plastome data reported in this study will contribute to further studies of phylogeny and conservation of *Phaius* species.

*Phaius hainanensis* C. Z. Tang et S. J. Cheng is a critically endangered orchid with significant ornamental value. It is only known from a single natural distribution area in Hainan Province in China (Chen et al. 2009). Due to habitat degradation, loss, and intrinsic factors of the species, *P. hainanensis* was listed as a wild plant with extremely small populations by the State Forestry Administration of China (Wade et al. 2016). With the popularity of Next-Seq technology, coupled with the moderate length and evolution rate of the chloroplast genome, chloroplast genome analysis has been used frequently for species evolution analysis, and for the establishment of conservation strategies for endangered species (Kuang et al. 2011; Li et al. 2019; Tang et al., 2021). To document genetic history of *P. hainanensis* and to contribute to its evolutionary systematics, we assembled and analyzed the plastome of *P. hainanensis* from Hainan Province, China.

The fresh leaves of *P. hainanensis* were collected from the Orchid Conservation Center of Yunnan Yelantang Biotechnology Co., Ltd, Yunnan Province, China (24.820301°N, 102.643764°E). The voucher specimens under the number Y. Zhang & Z. Zhang 2020015 were deposited in the herbarium of Xishuangbanna Tropical Botanical Garden, Chinese Academy of Sciences (HITBC, http://hitbc.xtbg.ac.cn, contact Jianwu Li, ljw@xtbg.org.cn). Total genomic DNA was extracted using TiangenDNA kit (TIANGEN, Beijing, China) following the manufacturer instructions, and sequenced by the Illumina NovaSeq platform (Illumina, San Diego, CA) at Personal Biotechnology Co., Ltd (Shanghai, China). The DNA of *P. hainanensis* was deposited in the Laboratory of Orchids biodiversity and conservation in Xishuangbanna Tropical Botanical Garden, Chinese Academy of Sciences with collection number xtbgorchid20200139 (contact with Dr. Yan Luo, luoyan@xtbg.org.cn). The total 3.7 G bases of raw data were generated and trimmed, and then were used to assemble the plastome using a toolkit GetOrganelle version 1.7.1a (Jin et al. 2020). The raw data were deposited in GenBank with associated number, BioProject PRJNA700130, SRA SRS8209487, and Bio-Sample SAMN17825178. The assembled plastome was annotated by the web server CPGAVAS2 (Shi et al. 2019). The *tRNA* genes were verified by tRNAscan-SE version 2.0.3 (Lowe and Chan 2016). The annotated plastome of *P. hainanensis* was deposited in GenBank under accession number MW463050.

The plastome of *P. hainanensis* is 158,314 bp in length and has a typical quadripartite structure. The genome is consisted of a large single copy region (LSC: 86,700 bp), a small single copy region (SSC: 18,452 bp), and two inverted repeat regions (IRs: 26,581 bp). The total GC content of plastome is 36.9%, and the corresponding values of LSC, SSC, IR regions are 34.7%, 29.9%, and 43.0%, respectively*. Phaius hainanensis* plastome encoded 114 unique genes, including 80 protein-coding, 30 *tRNA*s, and four *rRNA*s. Seventeen genes have a single intron while the two genes (*clpP* and *ycf*3) have two introns.

For the phylogenetic analysis, 73 protein-coding sequences from the plastome of 20 species classified to subfamily Epidendroideae in the Orchidaceae, were exported using Geneious Primer 2020 (Biomatters, Auckland, New Zealand), and aligned with MAFFT version 7.450 (Katoh and Standley 2013) and Mauve version 2.4.0 (Darling et al. 2004). The concatenated sequences were used for the phylogenetic analysis for the maximum likelihood (ML) and Bayesian inference (BI) analyses. The ML analysis was performed with IQ-TREE version 2.0.5 (Minh et al. 2020) with the best-fit model TVM + F+R2 automatically selected by ModelFinder (Kalyaanamoorthy et al. 2017). Branch support was evaluated by 1000 bootstrap replicates. The BI tree was inferred with MrBayes version 3.2.4 (Ronquist et al. 2012) in PhyloSuite (Zhang et al. 2020). The topologies obtained by the ML and BI analysis were consistent (Figure 1). Phylogenetic analysis suggested that *P. hainanensis* is sister to *P. tancarvilleae*, forming a monophyletic clade closely related to the genus *Calanthe* (Figure 1), corresponding with the phylogenetic relationships in the tribe Collabieae inferred by Xiang et al. (2014). The plastome data in this study will contribute to further studies of phylogeny of the tribe Collabieae.

**Figure 1.**
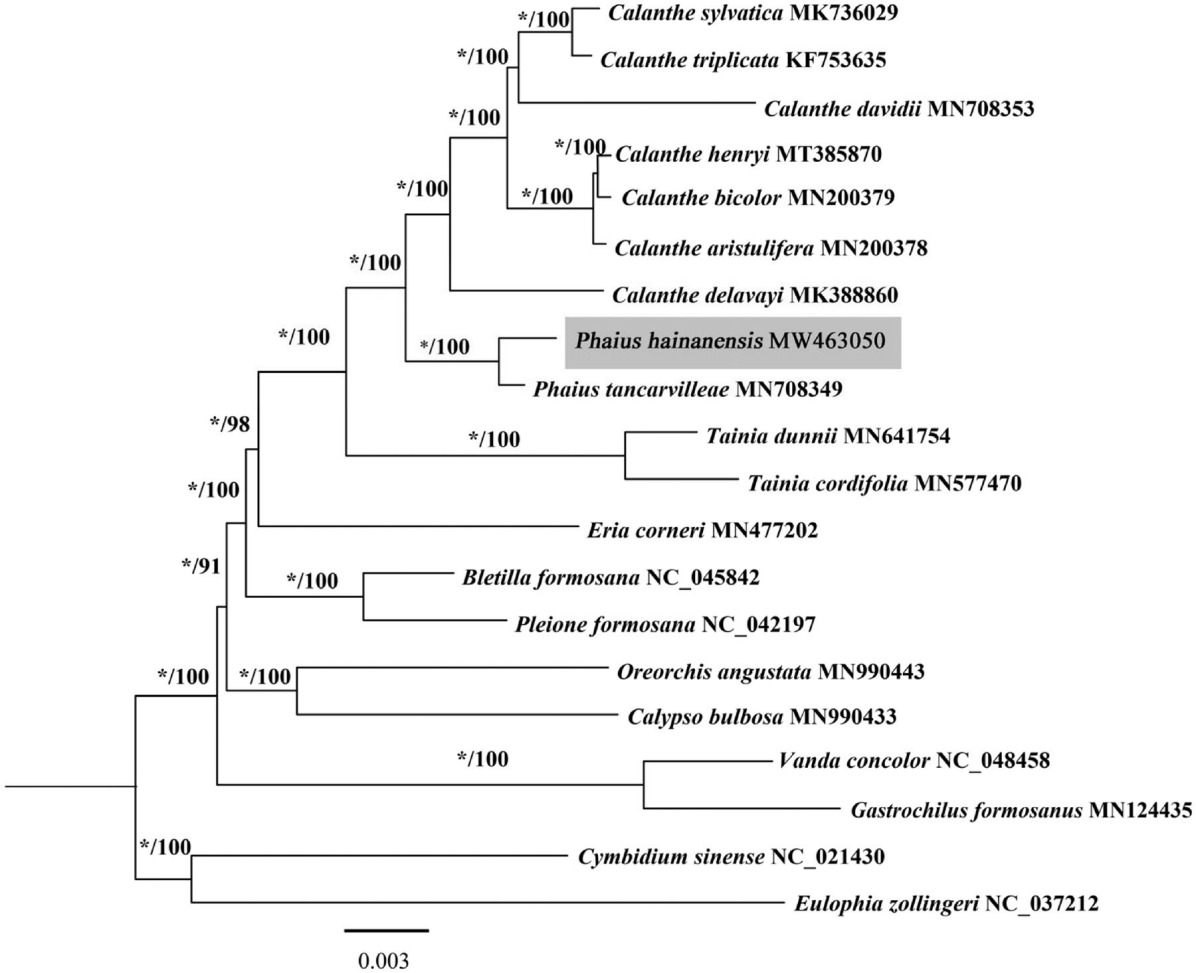
Phylogenetic tree conducted using maximum likelihood and Bayesian inference methods based on 73 protein-coding chloroplast genes from 20 Orchidaceae species. The numbers above branches represent posterior probability/bootstrap percentages based on 1000 replicates, ‘*’ indicates node is with support value 1.00.

## Data Availability

The genome sequence data that support the finding of this study are openly available in GenBank of NCBI at (https://www.ncbi.nlm.nih.gov) under the accession no. MW463050 (https://www.ncbi.nlm.nih.gov/nuccore/MW463050). The associated BioProject, SRA, and Bio-Sample number are PRJNA700130, SRS8209487, SAMN17825178, respectively (https://www.ncbi.nlm.nih.gov/bioproject/700130).
